# Parasitic nematode secreted phospholipase A_2_ suppresses cellular and humoral immunity by targeting hemocytes in *Drosophila melanogaster*

**DOI:** 10.3389/fimmu.2023.1122451

**Published:** 2023-03-15

**Authors:** Sophia C. Parks, Ogadinma K. Okakpu, Pakeeza Azizpor, Susan Nguyen, Stephanie Martinez-Beltran, Isaiah Claudio, Kyle Anesko, Anil Bhatia, Harpal S. Dhillon, Adler R. Dillman

**Affiliations:** ^1^ Department of Nematology, University of California, Riverside, CA, United States; ^2^ Metabolomics Core Facility, IIGB, University of California, Riverside, CA, United States

**Keywords:** sPLA2, *Steinernema carpocapsae*, immune modulation, *Drosophila*, host-parasite interactions

## Abstract

A key aspect of parasitic nematode infection is the nematodes’ ability to evade and/or suppress host immunity. This immunomodulatory ability is likely driven by the release of hundreds of excretory/secretory proteins (ESPs) during infection. While ESPs have been shown to display immunosuppressive effects on various hosts, our understanding of the molecular interactions between individual proteins released and host immunity requires further study. We have recently identified a secreted phospholipase A2 (sPLA_2_) released from the entomopathogenic nematode (EPN) *Steinernema carpocapsae* we have named Sc-sPLA_2_. We report that Sc-sPLA_2_ increased mortality of *Drosophila melanogaster* infected with *Streptococcus pneumoniae* and promoted increased bacterial growth. Furthermore, our data showed that Sc-sPLA_2_ was able to downregulate both Toll and Imd pathway-associated antimicrobial peptides (AMPs) including drosomycin and defensin, in addition to suppressing phagocytosis in the hemolymph. Sc-sPLA_2_ was also found to be toxic to *D. melanogaster* with the severity being both dose- and time-dependent. Collectively, our data highlighted that Sc-sPLA_2_ possessed both toxic and immunosuppressive capabilities.

## Introduction

Nematode parasitism is an important global health and agricultural issue, responsible for significant morbidity and mortality to humans, illness to livestock, and a reduction of global crop yields (1–3). Parasitic nematodes have ravaged human populations, with over 1.5 billion people being infected by soil-transmitted helminths alone (4). This issue is further compounded by recurrent reinfection and emerging drug resistance. Parasitic nematodes are thus very effective parasites, capable of evading and compromising the immune response of various hosts including insects and vertebrates (5–7). Despite the vast clinical knowledge on parasitic nematode infections, our understanding of the mechanisms that underlie helminths’ ability to modulate host immunity remains incomplete. By elucidating the molecular mechanisms of parasitic nematode immunomodulation, more effective anti-helminthic therapeutics can be produced, as well as potential therapeutics for treating human immune pathologies such as autoimmune diseases.

Parasitic nematodes are able to evade and alter host immunity *via* their release of excretory/secretory proteins (ESPs). ESPs consist of a variety of proteins that have effects ranging from metabolic breakdown of host tissue to immunomodulatory capabilities. Immunomodulatory proteins are able to promote the survival of parasitic nematodes during infection by strategically altering the activation of the host immune response (8). Characterization of individual proteins has remained challenging due to the technical obstacles of vertebrate model systems for testing hypotheses of potential effector proteins. Utilization of insect model systems however, has resulted in molecular characterization of individual proteins found in EPN ESPs (9). Due to the high homology EPN ESPs have with nematode parasites of vertebrates such as *Strongyloides stercoralis*, the molecular mechanisms of their ESPs are likely conserved (10–12). Effector proteins of the EPN *Steinernema carpocapsae* were assessed using the host *Drosophila melanogaster* due to its highly conserved innate immune system, with key immune signaling pathways and transcription factors resembling those in mammals (13). The *D. melanogaster* immune response includes a humoral and a cellular component (14, 15). The humoral immune response activates genes needed to synthesize and secrete antimicrobial peptides (AMPs) from the fat body into the hemolymph (16–18). Cellular immune responses are regulated by hemocyte function (19). Hemocytes regulate several cellular response mechanisms including cell aggregate formation, phagocytosis, melanization, and encapsulation which aid in fighting infections (20, 21). Melanization occurs after the production of phenoloxidase (PO) *via* up-regulation of prophenoloxidase (22, 23). PO serves as a catalyst for melanization by mediating the oxidation of mono- and di-phenols to quinones and is then followed by subsequent polymerization to form antimicrobial melanin (24, 25). This process ultimately results in the generation of reactive oxygen species (ROS) lethal to microbes (24). Activation of the immune response is generally regulated by two NF-kB signaling pathways: Toll and Imd which are similar to human toll-like receptors (TLR) and tumor necrosis factor (TNF) signaling respectively (14). Activation of these pathways is thought to be pathogen specific and depend on external cellular properties such as cell wall composition. Systemic production of specific AMPs *via* the humoral response is dependent on whether the Toll or Imd pathway is activated (26–28). EPNs must evade, suppress, and/or modulate the insect immune response by releasing effector proteins during infection to survive and complete their life cycle.

One family of effector proteins identified in the EPN *S. carpocapsae* was the secretory phospholipase A2 proteins (sPLA_2_) (29). The sPLA_2_ proteins are low molecular weight (13-19 kDa), and are Ca2+-dependent secretory enzymes that consist of 12 groups (30). In insects, PLA_2_ function has been shown to play a role in digestive physiology, immunity, reproduction, and fat body function (31). Insect PLA_2_ components of venom have been shown to cause pathologies such as anaphylaxis by eliciting cellular membrane disruption, inflammation, cellular necrosis, apoptosis induction, neurotoxicity, and hemolysis (32). PLA_2_ function is characterized by the ability to cleave cellular, non-cellular, and exogenous phospholipids to generate the eicosanoid precursor arachidonic acid (AA) along with saturated, monounsaturated, and polyunsaturated fatty acids (PUFAs) (33, 34). PUFAs generated include ω-3 eicosapentaenoic acid (EPA) and docosahexaenoic acid (DHA), both of which are precursors of anti-inflammatory lipid mediators (35, 36). Free AA produced by PLA2s are oxygenated by cyclooxygenase (COX) to yield prostaglandins (PGs), and by lipoxygenases (LOX) to yield leukotrienes (LTs). Cytochrome P450 monooxygenase can also change a double bond in AA to an epoxide, leading to the production of epoxyeicosatrienoic acids (EETs) (37). Most terrestrial insects, however, lack AA-derived PUFAs, as their endogenous PLA2s cleave linoleic acid (LA) which can be converted to AA by desaturases and long chain fatty acid elongase (38, 39). The newly formed AA can then undergo further conversion to a PGH_2_, which possesses a five membered ring structure that is characteristic of PGs, before further conversion to PGs *via* cell specific enzymes. It has been reported that AA is not converted to PGH_2_ by COX in insects. It is instead converted to PGH_2_ *via* an insect peroxidase called peroxinectin (40, 41). PGH_2_ is then converted into cell specific PGs *via* cell specific enzymes, such as PGE_2_ synthase converting PGH_2_ into PGE_2_ (42). PGs are involved multiple physiological roles in insects such as eggshell production, signaling of actin remodeling, regulation of actin bundle formation during oogenesis in *Drosophila*, and regulation of fascin localization to the nucleus (43–45). PGs play crucial roles in immune responses in insects by mediating the activation of hemocyte-spreading behavior involved in phagocytosis, nodulation, and encapsulation (37, 46).

This study characterizes the immunomodulatory effects of Sc-sPLA_2_ (gene L596_023809) on *D. melanogaster* against bacterial infection. Survival and bacterial proliferation were assessed after a one-time coinjection of Sc-sPLA_2_ and bacteria. Toxicity of the protein was also measured by administering a one-time dose of Sc-sPLA_2_ to *D. melanogaster*. To understand the mechanisms contributing to immunosuppression, readouts of downstream immune responses were assessed, including AMP production, PO activity, and phagocytosis. Metabolomic analyses were conducted on hemolymph of flies treated with Sc-sPLA_2_ to screen for changes in lipid metabolite and fatty acid composition. A cell lysis assay was used to determine whether toxicity was linked to lysis of host cell membranes, and hemocyte perfusion was performed to see if hemocyte circulation was affected by treatment with Sc-sPLA_2_.

## Results

### Sc-sPLA_2_ has a toxic and immunomodulatory effect

**Figure 1:**
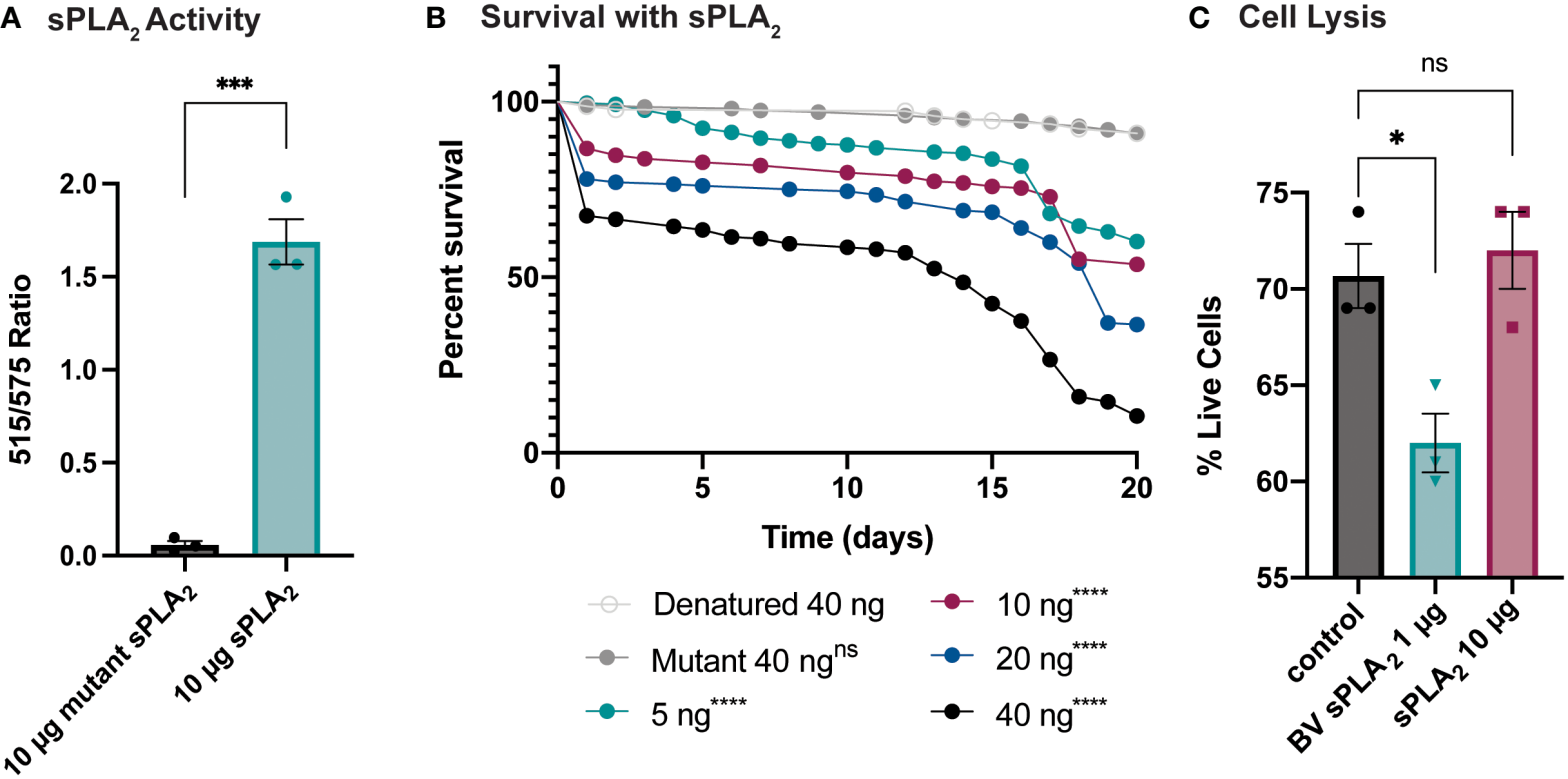
Survival rate of sPLA_2_-only injected flies shows a dose-dependent toxic effect not caused by cell lysis. **(A)** *In vitro* activity data of Sc-sPLA_2_ and mutant Sc-sPLA_2_ (HH82-83QQ) at 10 µg each. Fluorescent emission intensity was measured at 515 and 575 nm and recorded as a ratiometric value. Negative control was subtracted as background from both absorbance values before calculating the ratio. Substrate used was a Red/Green Bodipy labeled PC. Experiment was done in triplicate. Statistics shown as unpaired t-test, error bars depict mean with SEM, p=0.0002, 4 degrees of freedom, n=3. **(B)** To measure the toxicity of the *S. carpocapsae* sPLA_2_, 5–7-day old male flies were injected with various concentrations of protein and their survival was monitored for 20 days. Denatured protein shows no toxicity, and the intact protein shows a dose-dependent toxic effect with 40 ng showing the most significant toxicity. Survival curves n≥180. Log-rank test p-value significance compared to denatured 40 ng indicated by asterisks on Kaplan Meier graphs. 5, 10, 20, and 40 ng p<0.0001. Median survival is undefined for denatured, mutant, 5, and 10 ng, 19 days for 20 ng, and 14 days for 40 ng. **(C)** Quantification of cell lysis was measured by % of live cells after staining with a Bio-Rad TC20 automated cell counter. Sc-sPLA_2_ showed no significant changes to the % of live cells, while bee venom sPLA_2_ had a significant reduction which indicated an increase in cell lysis. Reactions were done in triplicate. Statistics shown as ordinary one-way ANOVA with Dunnett’s multiple comparisons test p=0.0222, error bars depict mean with SEM, 8 degrees of freedom, n=3. All raw data available in **Supplemental Materials**. ns, not significant.

**Figure 2:**
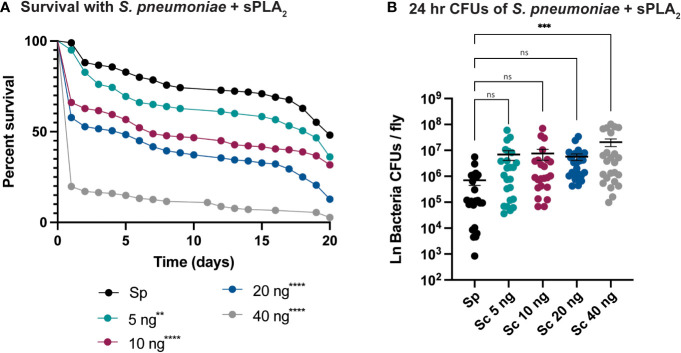
Sc-sPLA_2_ elicits a dose-dependent immunomodulatory effect on survival and 24-hour CFUs in *Streptococcus pneumoniae* and sPLA_2_ coinjections. **(A, B)** 5–7-day old male flies were coinjected with 2,000 cells of *S.p.* and various nanogram doses of sPLA_2_. **(A)** Their survival was monitored for 20 days, showing a significant reduction in the outcome of survival in all doses compared to the *S.p.* only injected flies. Log-rank test p-value significance indicated by asterisks on Kaplan Meier graphs, n≥180. All doses were compared to *S.p.*-only dose, 5 ng p=0.0067, 10, 20, and 40 ng p<0.0001. Median survival of 20 days for *S.p.*-only, 17 days for 5 ng, 7 days for 10 ng, 5 days for 20 ng, and 1 day for 40 ng. **(B)** CFUs were measured 24-hours after injection and the 40 ng dose shows a significant increase in microbe load compared to the *S.p.*-only control group. Statistics shown as ordinary one-way ANOVA with Dunnett’s multiple comparisons test p=0.0008 with 118 degrees of freedom. Error bars show mean+SEM, n≥24. When compared to the 40 ng dose, the 5 and 10 ng doses were not significant while 20 ng was significantly lower (p=0.0370). All raw data available in **Supplemental Materials**. ns, not significant.

An enzymatic assay was used to quantify the biological activity of recombinantly expressed Sc-sPLA_2_ and a catalytically inactive mutant Sc-sPLA_2_ (HH82-83QQ). Each protein was tested with a Red/Green BODIPY labeled phosphatidylcholine (PC) substrate, and fluorescence emission intensity was measured and reported as a ratiometric value. The mutant sPLA_2_ displayed significantly less activity than the wild type with a fluorescent 515/575 ratio close to 0, while the wild type displayed a fluorescent 515/575 ratio of over 1.5 (**Figure 1A**). Prior to assessing potential immunomodulatory phenotypes, a dose response for potential toxicity of Sc-sPLA_2_ was measured. Toxicity increased as the dose increased where 5 ng of Sc-sPLA2 elicited minimal toxicity with over 90% survival rate by day 5, while 40 ng showed only a 65% survival rate in the same time frame (**Figure 1B**). Denatured protein displayed no toxicity throughout the 20-day period post injection, while all doses of Sc-sPLA_2_ had an increase in toxicity after day 15 post injection (**Figure 1B**). To determine if the toxicity was linked to cell lysis, a cell lysis assay was performed with *D. melanogaster* Schneider 2 (S2) cells. Cells were treated with Sc-sPLA_2_ or bee venom sPLA_2_, which was screened for activity prior to experimentation to confirm enzymatic function (**Supplementary Figure 3**). The Sc-sPLA_2_ did not cause cell lysis, while the bee venom PLA_2_ showed an increase in cell lysis by significantly reducing the amount of live cells (8% reduction), showing that our findings are consistent with previous reports (**Figure 1C**) (47). After evaluation of toxicity, each dose of Sc-sPLA_2_ was then coinjected with 2,000 cells *S.p.* (LD_10_) where we observed a significant reduction in survival after a one-time dose over the course of 20 days (**Figure 2A**). Sc-sPLA_2_ significantly reduced the survival rate at each dose with the highest reduction observed at a dose of 40 ng which displayed a survival rate of only 20% after day 1 (**Figure 2A**). Microbial growth was also measured 24 hours post coinjection. We observed a significant increase in microbial load, approximately a 10-fold increase at 40 ng (**Figure 2B**). The mutant Sc-sPLA_2_ (HH82-83QQ) showed no change to the survival of the flies during coinjection (**Supplementary Figure 1**), confirming that the enzymatic activity of Sc-sPLA_2_ was responsible for the immunomodulatory phenotypes observed.

### Sc-sPLA_2_ suppresses specific downstream immune responses

**Figure 3:**
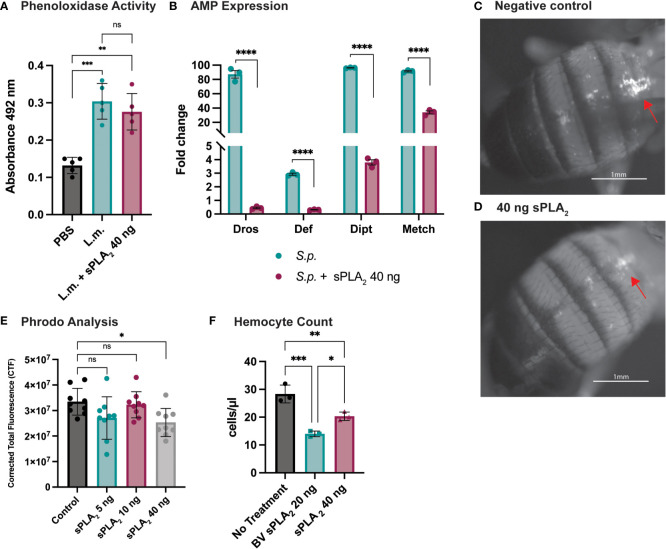
Specific downstream immune responses are affected by Sc-sPLA_2_. **(A)** Phenoloxidase activity was measured 6 hours after injection with either PBS control, 10,000 cells *Listeria monocytogenes*, a known melanizer, or *L.m.* plus protein. An increase in PO activity was observed in the bacteria injected group but was not altered by the presence of protein. Experiments were completed 5 times with 30 flies in each treatment group. Statistics shown as ordinary one-way ANOVA with Dunnett’s multiple comparisons test *L.m.* p<0.0001, 40 ng p=0.0003 with 14 degrees of freedom, error bars depict mean with SEM. **(B)** Antimicrobial peptide production was measured by quantitative PCR 24 hours after injection with *S.p.* or *S.p.* plus protein. Four different AMPs were measured, *Drosomycin* (Toll), *Defensin* (Imd), *Diptericin* (Imd), and *Metchnikowin* (Toll) were all decreased after protein injection. Statistics shown as 2-way ANOVA with a Tukey multiple comparisons test, p<0.0001 for all sets with 16 degrees of freedom. Experiments repeated 3 times with 15 flies in each group. **(C)** Phagocytic activity was measured with the pHrodo assay showing fluorescence once phagocytosed. pHrodo only injected flies show higher amounts of fluorescence. **(D)** Fluorescence is decreased in flies injected with 40 ng of sPLA_2_ protein. Representative images are depicted. **(E)** We found the 40 ng dose of sPLA_2_ protein to significantly reduce phagocytosis one hour post injection. The 5 and 10 ng doses are not significantly different from the 40 ng sPLA_2_ dose. Statistics shown as ordinary one-way ANOVA with Dunnett’s multiple comparisons test p=0.0243 with 35 degrees of freedom. Experiments were repeated 3 times with 3 flies per group. **(F)** We found that 40 ng dose of sPLA_2_ protein had a significant reduction of circulating hemocytes one hour post injection. Each experiment was repeated 3 times with 10 flies in each group for every treatment. Statistics shown as ordinary one-way ANOVA with Tukey’s multiple comparisons test. Asterisks indicating the following p-value cut offs: 0.05-0.033*, 0.033-0.002**, 0.002-0.0001*** and <0.0001****. ns, not significant.

To better understand how Sc-sPLA_2_ modulates immunity, we evaluated several readouts of immunity including PO activity and AMP production. PO activity serves as a catalyst for melanization. Flies were coinjected with Sc-sPLA2 and *Listeria monocytogenes*, a bacterium that elicits a robust disseminated melanization phenotype, to measure any changes to PO activity (24, 48). Treatment with Sc-sPLA_2_ showed no significant changes to PO activity compared to the Listeria-only group (**Figure 3A**). To further evaluate specific downstream immune responses, AMP production was measured 24-hours postinjection. The protein treatment significantly reduced expression of *defensin* (Imd), *metchnikowin* (Imd), *diptericin* (Toll), and *drosomycin* (Toll), suggesting a suppressive effect on the Toll and Imd pathways (**Figure 3B**) (27, 28).

Phagocytosis is another important downstream immune process that is regulated by the cellular branch in insect immunity (20, 21). Phagocytic activity in *D. melanogaster* was visualized and quantified *via* injection of fluorescently labeled conjugates of *E. coli* that fluoresce after exposure to the lysosome’s low pH environment. These conjugates were coinjected with Sc-sPLA_2_ to assess any changes in phagocytosis. We found that a one-time dose of 40 ng of Sc-sPLA_2_ was able to significantly decrease phagocytosis activity 1-hour post injection (**Figures 3C, D**). Flies with the 40 ng dose had an average CTF of about 2.5*10^7^, while the negative control had a CTF of about 3.3*10^7^. 5 ng and 10 ng doses showed no significant effect on fluorescent change (**Figure 3E**). We evaluated if Sc-sPLA_2_ targeted circulating hemocytes by measuring hemocyte concentration 1-hour post injection with enzyme. Flies injected with 40 ng of Sc-sPLA_2_ had an average hemocyte concentration of 20 cells/μl. The negative control group (PBS) had an average concentration of 28 cells/μl, and the positive control group (20 ng bee venom sPLA_2_) had an average concentration of 14 cells/μl (**Figure 3F**). This result ultimately shows that Sc-sPLA_2_ is having a suppressive effect on the cellular and humoral responses of *D. melanogaster* immunity by targeting circulating hemocytes.

### Sc-sPLA_2_ displays exponentially higher activity with PLPE and AA

**Figure 4:**
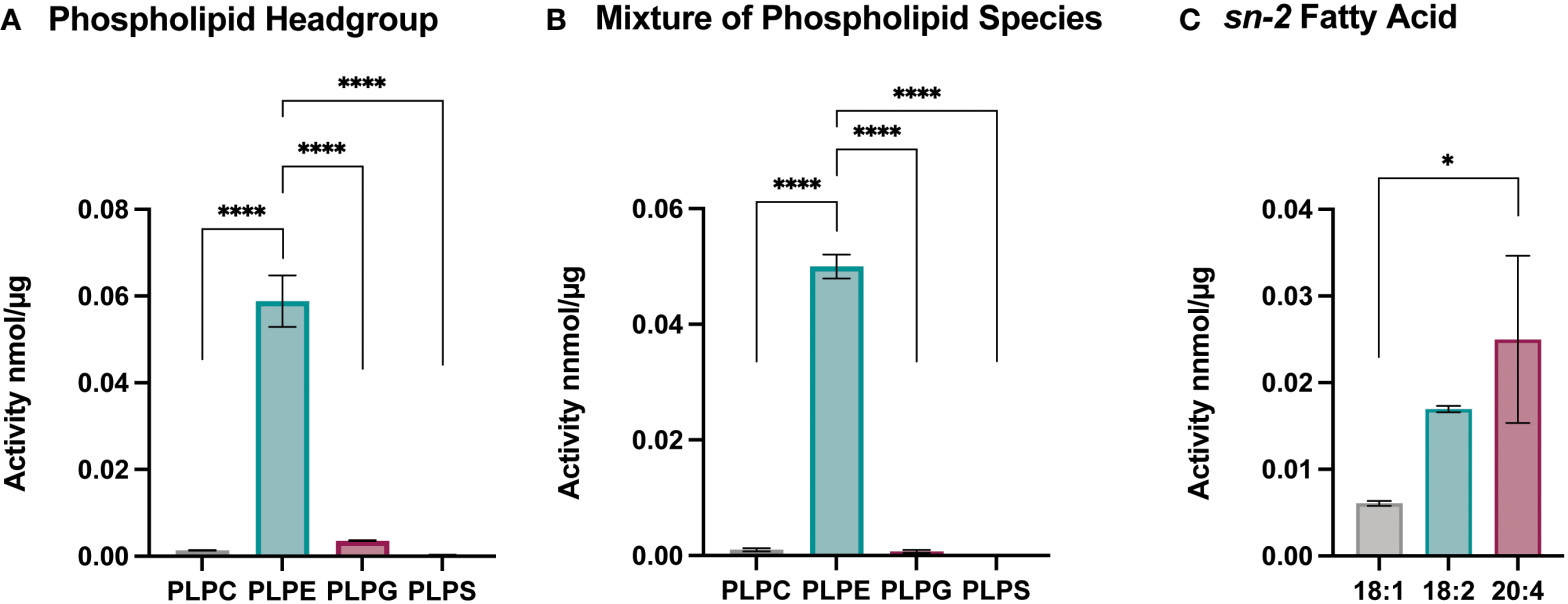
Enzymatic activity assays show sPLA_2_ prefers PLPE. **(A)** Enzymatic activity of Sc-sPLA_2_ towards 100 µM of PLPC, PLPE, PLPG and PLPS. Statistics shown as ordinary one-way ANOVA with Tukey’s multiple comparisons test. PLPC vs PLPE, PLPE vs. PLPG, and PLPE vs. PLPS p<0.0001 with 11 degrees of freedom. **(B)** Enzymatic activity of Sc-sPLA_2_ towards 100 µM mixture (20 µM each) of PLPC, PLPE, PLPG and PLPS. Statistics shown as ordinary one-way ANOVA with Tukey’s multiple comparisons test. PLPC vs PLPE, PLPE vs. PLPG, and PLPE vs. PLPS p<0.0001 with 11 degrees of freedom. **(C)** Enzymatic activity of Sc-sPLA_2_ towards 100 µM PLPE species with an *sn-*2 fatty acid position of OA (18:1), LA (18:2), and AA (20:4). Statistics shown as ordinary one-way ANOVA with Tukey’s multiple comparisons test. 18:1 vs. 20:4 p=0.0142 with 8 degrees of freedom. Negative control values were subtracted from each reaction. Experiments were done in triplicate. Error bars depict mean with SEM.

To better understand the effect of Sc-sPLA_2_ on lipid metabolism and which phospholipid sources were a preferred target for this enzyme, we utilized lipidomics by performing a high-throughput mass spectrometric based assay (49). This assay revealed the *in vitro* activity of Sc-sPLA_2_ towards both natural and synthetic membrane phospholipids in mixed micelles. First, we explored preference of Sc-PLA_2_ for phospholipid head group by using four major phospholipid head groups for the *sn-3* position which included phosphoethanolamine (PE), phosphoserine (PS), phosphoglycerol (PG), and phosphocholine (PC). For the *sn-1* position we utilized palmitic acid due to its ability to be produced *de novo* in *Drosophila* (50). Previous studies showed that the *sn-1* fatty acid did not affect the activity of PLA2s and thus we did not conduct optimization for that position (49). For optimization and head group studies we utilized linoleic acid (LA) at the *sn-2* position since it is the most abundant PUFA in *D. melanogaster* (50). Reactions were run for 30 minutes as it was determined in previous studies to be the most optimal time for multiple PLA_2_s (49). Surfactant concentration and enzyme concentration optimization reactions were conducted to determine the conditions to use for downstream experimentation (**Supplementary Figure 2**). For determining Sc-sPLA_2_ preference for phospholipid head groups, we used the lipid substrate 1-palmitoyl-2-linoleoyl-sn-glycero-3-phosphox where “x” represents one of the four major lipid head groups. Thus, the lipid headgroup substrates utilized for the reaction were PLPE, PLPS, PLPG and PLPC. The experiment showed that activity towards PLPE by Sc-sPLA_2_ was exponentially higher than all other headgroups (**Figures 4A, B**). Interestingly, PE abundance has been linked to Toll pathway expression in *D. melanogaster* (51). We performed subsequent experiments using lipid substrates with the PE headgroup to determine Sc-sPLA_2_ fatty acid preference at the *sn-2* position. The fatty acids used were oleic (OA), LA, and arachidonic acid (AA). OA was selected as it is an 18-carbon fatty acid with high abundance in *D. melanogaster* and can be converted to LA (52, 53). LA and AA are precursors to immunomodulating eicosanoids with AA being the most common precursor in mammals (33, 36, 54). We found that Sc-sPLA_2_ displayed high activity to both LA and AA in comparison to OA (**Figure 4C**). Overall, these data illustrate that Sc-sPLA_2_ displays high activity with lipid species that have downstream effects on immunity.

### Sc-sPLA2 alters fatty acid composition *in vivo*

**Figure 5:**
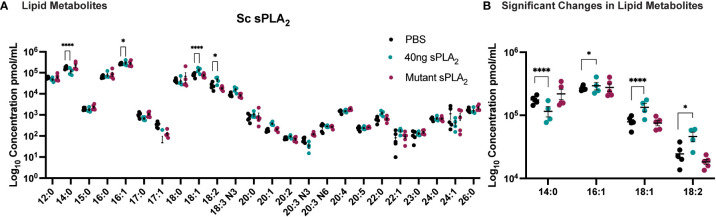
Injection of 40 ng recombinant Sc-sPLA_2_ induced significant changes in lipid metabolites. **(A)** Lipid panel consisted of 33 fatty acids but only 24 were detected in the fly hemolymph samples. **(B)** A reduction of myristic acid (MA-14:0) compared to both PBS (p<0.0001) and the mutant control (p<0.0001). Sc-sPLA_2_ generated more oleic acid (OA-18:1) compared to both PBS and the mutant control (to PBS p<0.0001, to mutant p=0.0023), and linoleic acid (LA -18:2) compared to PBS (p=0.0414) and increased palmitoleic acid (PA-16:1) in comparison to the PBS control (p=0.0256). There were no differences between the mutant and PBS control groups. Experiments were repeated 5 times with 200 flies per treatment group. Error bars show mean + SEM with statistics shown as multiple unpaired t-tests.

To further elucidate the underlying molecular mechanisms of Sc-sPLA_2_’s immunomodulatory effects, we used mass spectrometry to analyze the hemolymph of protein-injected flies. A targeted approach allowed for identification of known fatty acids and lipid metabolites that were altered after treatment with the protein. sPLA_2_ activity produces lipids that can have roles as immune mediators and thus we anticipated this experiment would identify fatty acids and lipid metabolites with a significant role in insect immunity (30). The lipid panel for the mass spectrometry analysis consisted of 33 lipids. 24 fatty acids were detected in the fly hemolymph, including C20, C22, C23, C24, C26 fatty acids (**Figure 5**). When comparing the Sc-sPLA_2_ treatment group to the PBS control, we observed an increase in LA, OA, and palmitoleic acid (PA-16:1), with a decrease in myristic acid (MA-14:0). When compared to the mutant control group, Sc-sPLA_2_ elicited the same significant changes except it did not significantly change PA, and no significant changes in lipid profiles between the PBS and mutant control groups were observed (**Figure 5**). Out of the downstream oxylipin metabolite library used for further analysis we saw significant abundance of 17 different lipid metabolites, with Sc-sPLA_2_ causing a significant reduction of 9, (10)-, and 12, (13)-EpOME at 12-hours post-injection (**Supplementary Figure 4**).

## Discussion

It has been well established that sPLA_2_ activity plays an important role in immune response by cleaving PUFAs such as AA from glycerophospholipids resulting in production of downstream immunomodulatory eicosanoids (33, 34). While this process is well defined in mammals, the presence of lipid signaling in insect immunity has not been validated and has even been disputed due to their lack of C20 and C22 PUFAs necessary for eicosanoid production (55). It has been recently reported however, that insects are able to generate eicosanoids and their precursor AA by converting cleaved LA into AA for downstream eicosanoid production (38, 54). With a potential mechanism in place for lipid signaling mediated immunity in insects, we evaluated the role of an sPLA_2_ from a parasitic nematode, Sc-sPLA_2_, in host immunomodulation to bacterial infections in *D. melanogaster*.

We hypothesized that Sc-sPLA_2_ would display immunosuppressive effects on its host since it is secreted by *S. carpocapsae* infective juveniles (IJs) during infection. sPLA_2_ enzymes are notable for eliciting immunostimulatory responses *via* downstream production of proinflammatory eicosanoids from arachidonic acid (33). However, they are also able to cleave PUFAs such as EPA and DHA which are then converted to downstream anti-inflammatory mediators, indicating that sPLA_2_ enzymes can also have immunosuppressive capabilities (33). In addition to immunomodulatory effects, PLA_2_ enzymes have been reported to display toxic effects on hosts. This is facilitated by necrotic cell lysis *via* enzymatic cleavage of the phospholipid cell membrane by PLA_2_s, resulting in loss of cell membrane integrity and release of cellular components (47, 56). Sc-sPLA_2_ was able to display a dose dependent toxic effect in *D. melanogaster* at a low dose of Sc-sPLA_2_ (5 ng). These flies had around a 95% survival rate by day five in comparison to the higher dose (40 ng) that displayed a 65% survival rate. After day five toxicity had a slow increase for all doses up until day 15 where another notable increase in toxicity occurred resulting in decreased survival rates. This highlighted that the enzyme’s toxic effects on the host were both time- and dose-dependent. We found no significant change in the amount of cell lysis of S2 cells in comparison to the negative control, indicating that the toxic effects are not caused by cell lysis, but perhaps because of other cell death mechanisms such as apoptosis and necroptosis. The fact that Sc-sPLA_2_ elicits an immunosuppressive phenotype at a 5 ng dose shows significant importance as this is a physiologically relevant dose. Twenty IJs of *S. carpocapsae* secrete approximately 10 ng of crude ESPs in 24 hours (29). The ES protein composition of *S. carpocapsae* is approximately 500 proteins, with Sc-sPLA_2_ being just one component. With enough IJs however, and the mixture of multiple immunomodulatory proteins, it is likely that Sc-sPLA_2_ aids in overcoming the host immune response in a natural infection.

We evaluated the effects of Sc-sPLA_2_ on downstream immunity in the fly. Fly immunity starts with pathogen specific recognition by the toll and imd pathways, which then leads to either a cellular immune response by specialized hemocytes, or a humoral immune response *via* production of Toll- or Imd-specific AMPs secreted from the fat body (19, 26). Melanization is independent of the Toll and Imd pathways and is dependent on the proPO-PO cascade (22, 23). Our findings showed that Sc-sPLA_2_ had no effect on PO activity but caused a reduction in the expression of the AMPs *Metchnikowin, Diptericin, Defensin* and *Drosomycin* and significantly reduced phagocytosis at a one-time dose of 40 ng. There was not a significant effect on phagocytosis at 5 and 10 ng (**Figure 3E**). We speculate that this was due to the time point for observing fluorescence of the assay being too early for the lower doses to generate a significant difference. Methods for the pHrodo Red *E. coli* BioParticles conjugate state that fluorescence can be observed after 30-60 minutes, with many experiments opting to observe fluorescence generally after 2 hours. To further evaluate how Sc-sPLA_2_ specifically elicited these immunomodulatory phenotypes, we measured hemocyte circulation 1 hour post injection with 40 ng of Sc-sPLA_2_ and found a significant reduction in hemocyte concentration. This highlighted that the enzyme did in fact trigger cell death. We opted to observe effects one hour post injection to see how early introduction of the sPLA_2_ begins to affect the immune response. The effects at this early time point also reinforce that at least some immunosuppressive effects are observed prior to lethal toxicity as flies are still all alive at one hour post injection. Overall, these findings suggest that the Sc-sPLA_2_ suppresses both the Toll and Imd pathways along with cellular immune responses by targeting circulating hemocytes.

We utilized lipidomics for a mass spectrometry-based high-throughput assay to determine the preference of Sc-sPLA_2_ for specific lipid targets *in vitro*. These data would provide some insight regarding the lipids Sc-sPLA_2_ may target during a natural infection by *S. carpocapsae* in insects. Our data showed that Sc-sPLA_2_ had exponentially higher activity with PE as a substrate than the other lipid headgroups. This is significant as PE is the most abundant phospholipid for cellular membranes in *D. melanogaster* (51, 57). Displaying significant catalytic activity against PE lipid substrates is likely advantageous for Sc-sPLA_2_ in terms of modulating insect immunity. It is important to note that the Enzchek Bee venom sPLA_2_ also displayed its highest levels of activity with the PE substrate as opposed to PC (**Supplementary Figure 3**). The Enzchek activity kit utilizes PC as the commercial substrate, but our experiments show that it could be more advantageous to use PE as the substrate for commercial activity kits. We also used the novel mass spectrometry-based high-throughput assay to determine preference *in vitro* for fatty acid side chains at the sn-2 position. The *in vitro* assay showed that Sc-sPLA_2_ displayed highest activity with LA and AA at the *sn-2* position. Activity against OA was measurable but noticeably lower than activity against LA and AA. It is strategic for Sc-sPLA_2_ to display significant activity with OA as it can be converted to the eicosanoid precursor LA (52). Linoleic acid is the most abundant PUFA in insects such as *D. melanogaster* and like AA, it is a precursor to generating eicosanoids involved in downstream immune responses (36, 50). Arachidonic acid can generate pro-inflammatory eicosanoids in its omega-6 form, and anti-inflammatory eicosanoids in its omega-3 form. The higher activity levels displayed against these two PUFAs illustrates that Sc-sPLA_2_ could potentially be suppressing immunity by generating downstream immunosuppressive lipid metabolites, or by interfering with host endogenous sPLA_2_ activity. Little is known about endogenous sPLA_2_ activity in fly immunity.

We collected fly hemolymph after injection of Sc-sPLA_2_ to perform mass spectrometry to determine fatty acid and lipid metabolite composition 12-hours postinjection. Fatty acid composition postinjection showed an increase in PA, OA, and LA, and a decrease in MA. An increase in OA and LA is consistent with the high activity levels that Sc-sPLA_2_ displayed against phospholipids with these fatty acid side chains. Sc-sPLA_2_ increasing PA in the hemolymph may play a role in the downstream immunosuppressive phenotypes observed, as increased PA was shown to elicit anti-inflammatory effects in animal models (58). Our data showed there was an increase in PA by Sc-sPLA_2_ when compared to the PBS control, but there were no significant changes in PA compared to the mutant control group. A likely explanation is that the 12-hour time point was long enough for the low activity of the mutant to generate enough PA to affect the comparison. The mutant enzyme had low enough activity not demonstrate any immunomodulatory phenotypes but was still able to have low activity levels detected by the Enzchek activity assay. The data also showed a decrease in free MA that could be linked to the high activity demonstrated to the major lipid headgroup PE by Sc-sPLA_2_. PE is 50% of cellular membrane phospholipids in *D. melanogaster*, and MA is utilized to anchor proteins to the cellular membrane (57, 59). It is possible that disruption of the cellular membrane *via* cleavage of PE, could lead to free MA to be utilized in myristoylation and sidechain palmitoylation to anchor more proteins to the disrupted membrane for preserving stability and cellular function. This would then result in a reduction of free MA in the hemolymph. Myristic acid is known to play a direct role in two classes of protein fatty acid acylation: N-terminal myristoylation and side-chain palmitoylation (60). This promotes anchoring of proteins to the cell membrane (59). The protein substrates that are products of acylation carried out by Myristoyl–CoA: protein N-myristoyltransferase (NMT) include those that are key components of intracellular signaling (61). Palmitic acid has direct impact in immunity as it has been shown in animal models to decrease expression of proinflammatory markers and adipokines(58, 62–64). Palmitic acid suppresses the expression of monocyte chemoattractant protein 1 (MCP-1) and TNF-α in adipose tissue suggesting the lipid has downstream anti-inflammatory effects (62, 64). The fatty acids changed by Sc-sPLA_2_ each demonstrate a role in the immune response *via* lipid signaling or potential other downstream mechanisms, thus implicating several pathways the enzyme could be interfering with to suppress immunity other than hemocyte reduction. In addition to assessing fatty acid composition, we also analyzed the hemolymph for any downstream changes to lipid metabolite composition. Findings showed that Sc-sPLA_2_ treated flies had a reduction in 9,(10)-EpOME and 12,(13)-EpOME. These lipid metabolites are synthesized by activated neutrophils in mammals and are known low-level stimulators of respiratory burst, a process that occurs during phagocytosis (65–67). LA is a potent inducer of respiratory burst but is increased in the hemolymph after enzyme treatment (67). Soluble metabolites from epoxide hydrolase (DiHOMEs) are also directly responsible for respiratory burst inhibition, but there is no change in DiHOMEs after enzyme treatment (67). This information combined with previous studies that showed 9,(10)-EpOME and 12,(13)-EpOME to suppress immune response in *Spodoptera exigua*, make it likely their reduction by Sc-sPLA_2_ is a byproduct from the enzyme triggering cell death of hemocytes in the hemolymph (68). Overall, these data suggest that the molecular effects underlying sPLA_2_ suppression of the cellular immune response is *via* targeting of circulating hemocytes. Future studies on how Sc-sPLA_2_ directly affects hemocyte proliferation and morphology can help further elucidate mechanistically how the enzyme is reducing hemocyte circulation.

In summary this study showed that Sc-sPLA_2_ experimentally dampened the immunity of *D. melanogaster* by suppression of phagocytosis and both the toll and imd pathways. In addition to immunomodulation, Sc-sPLA_2_ also displayed dose-dependent toxicity to the host that was not elicited by cell lysis. We hypothesize that the lipids being cleaved by the PLA_2_ enzyme are from hemocytes which disrupts their ability to recognize and phagocytize cells, while producing a toxic molecular product to the host. The change in the lipid composition as a result of this process could also be disrupting the lipid signaling processes, leading to further suppression in immunity such as reduced toll and imd activation. Further elucidating the specificity of the molecular mechanisms affected by Sc-sPLA_2_ can continue to validate the presence of lipid signaling in *D. melanogaster* immunity, which would improve the tools available for biomedical research and further enhance the translation of fly research in addressing inflammatory and infectious diseases.

## Methods

### Plasmid construction

A 414 -bp DNA fragment of Sc-sPLA_2_ gene L596_023809 was amplified by PCR using primers 5’ – ACCATCATCACCACAGCCAGGGCAAACTTATCAAGAAGAATGTCG – 3’ (forward primer) and 5’ – TTAAGCATTATGCGGCCGCATTACGCGTGGAAATCGAGC – 3’ (reverse primer) in which a *Bam*HI site at the 5′ end and a *Hind*III site at the 3′ end was introduced for cloning it into a pETDuet-1 vector. The mutant Sc-sPLA_2_(HH82-83QQ) had two histidine amino acid sequences at positions 82 and 83, mutated to glutamine. The mutant was synthesized, optimized and inserted into a pETDuet-1 vector utilizing a *Bam*HI site at the 5′ end and a *Hind*III site at the 3′ position. The mutant construct was generated by Bio Basic Inc.

### Recombinant protein expression and purification

Sc-sPLA_2_ and mutant Sc-sPLA_2_ (HH82-83QQ) were recombinantly expressed using *E. coli* BL21 DE3 cells in LB media for 24 hours after induction with IPTG. Sc-sPLA_2_ was purified from inclusion bodies with Thermo Scientific™ HisPur™ Ni-NTA Resin *via* gravity filtraticon. The protein was refolded with a 24-hour dialysis against a 20 mM Tris, 1.0 M Urea, 300 mM NaCl, and 5% glycerol pH 8.0 buffer. After refolding the protein was dialyzed once more for 24 hours and stored in a 20 mM Tris, 300 mM NaCl, and 5% glycerol pH 8.0 buffer. Mutant Sc-sPLA_2_ (HH82-83QQ) was first purified with Thermo Scientific™ HisPur™ Ni-NTA Resin *via* gravity filtration. The protein was dialyzed against a 20 mM Tris pH 8.0 buffer for 24 hours. Further purification was conducted with FPLC using a Mono Q™ anion exchange column, after which the protein was isolated using size exclusion and stored in a 20 mM Tris, 300 mM NaCl, and 5% glycerol pH 8.0 buffer. Both Sc-sPLA_2_ and mutant Sc-sPLA_2_ (HH82-83QQ) presence were confirmed using SDS-PAGE. Concentrations were measured using Invitrogen™ Qubit™ Protein and Protein Broad Range (BR) Assay Kits, and the proteins were flash frozen with liquid nitrogen and stored at −80°C.

### Protein activity assay

Enzymatic activity of Sc-sPLA_2_ and mutant Sc-sPLA_2_ (HH82-83QQ) was assessed utilizing the EnzChek™ Phospholipase A2 Assay Kit. Each reaction contained 10 µg of protein and 50 µl 1.67 µM Red/Green BODIPY labeled phosphatidylcholine (PC) substrate for a total of 100 µl. Reaction time was 30 minutes at room temperature. Negative control was designated as buffer only plus the substrate. Emission intensity was measured at 515 and 575 nm with excitation at 460 nm, and the activity was recorded as a ratiometric value (515/575 nm). Negative control values at 515 and 575 nm were subtracted from the protein reactions before calculation of the activity ration. Reactions were triplicated.

### Lipidomics mass spectrometry assay

To determine activity preference of Sc-sPLA_2_ 1.0 ugs of the recombinantly expressed sPLA_2_ proteins was added to reactions that contained 100 uM of PLPC, PLPA, PLPG, PLPE, or PLPS, 400 uM of surfactant, 2.5 uM of 17:0 LPC and reaction buffer (20 mM Tris and 5 mM CaCl2 buffer pH 8.0). The reaction buffer was used to store the lipids and surfactant. For mixed phospholipid head group reactions, we used the same conditions as previously described for the enzyme, 17:0 LPC, surfactant, and buffer. For the lipid substrate however, all head groups were combined for a total concentration of 100 uM (20 uM for each different phospholipid headgroup). Enzymatic reaction was performed in a 96 well-plate using a Benchmark Scientific H5000-H MultiTherm heating shaker for 30 min at 28°C. Negative control was reaction buffer only with no protein, and positive control was 0.125 ugs of Enzchek Bee Venom sPLA_2_ to ensure the reaction is working as intended (these controls were used for all optimization and specificity reactions). Reactions were quenched with methanol/acetonitrile (80/20, v/v), and the samples were analyzed using the HPLC-MS system. Activity was reported as nmols/ug with subtraction of the negative control as background. Experiments were done in triplicate with error bars on the graph representing standard deviation. For determining activity preference for *sn-2* fatty acids, 1.0 ugs of the Sc-sPLA_2_ was added to a reaction of 100 uM phospholipid substrate, 400 uM surfactant, and 2.5 uM of 17.0 LPC. The phospholipid substrate used the experimentally determined preferred phospholipid head group PE. Each lipid substrate had a different *sn-2* fatty acid of either LA, OA, or LLA for a concentration of 100 uM in separate reactions. Enzymatic reaction was performed in a 96 well-plate using a Benchmark Scientific H5000-H MultiTherm heating shaker for 30 min at 28°C. Negative control was reaction buffer only with no protein, and positive control was 0.125 ugs of Enzchek Bee Venom sPLA_2_. Mass spectrometry analysis was conducted at the UC Riverside Core Facility. Experiments were done in triplicate with error bars on the graph representing standard deviation.

### UCR core facility QQQ lipidomics method

The targeted analysis was performed using a QQQ XEVO TQ-XS (Waters Corp., Milford, MA, USA) at the UC Riverside Metabolomics Core. The liquid chromatography-mass spectrometry (LC-MS) autosampler was maintained at 4°C prior to analysis. For the analysis, an injection volume of 2 μL of the extract was used. The separation was performed on the Waters XSelect CSH Phenyl-Hexyl column (3.5 μm, 3.0 × 100 mm (Waters Corp., Milford, MA, USA). The flow rate was maintained at 0.8 mL/min at 30 C. Mobile phase A consisted of ACN/water (95/5, v/v, pH=8.0) containing 25 mM AcNH4 and Mobile Phase B consisted of ACN/water (50/50, v/v, pH=7.5) containing 25 mM AcNH4. The gradient separation method was used as follows: 8 min (0–0.2 min 99% B; 0.2-3.0 min 99% B to 1%B, 3.0-3.8 min 1% B; 3.8-3.9 min 1% B to 99% B; 3.9-8.0 min 99% B. The MS data were acquired in multiple reaction monitoring (MRM) mode. The electrospray ionization was performed in positive ion mode. The source and desolvation temperatures were maintained at 150°C and 600°C, respectively. The desolvation gas was set to 1100 L/h and cone gas to 150 L/hr and the collision gas was set to 0.15 mL/min. All gases used were nitrogen, other than the collision gas which was argon. The capillary voltage was 1.5 kV. The data was normalized for relative abundance against the internal standard (LPC 17:0). Targeted data processing was performed with the open-source Skyline software (69).

### Fly stock/maintenance

Oregon R flies were grown on D2 glucose medium from Archon Scientific (Durham, North Carolina) and kept at 25°C with 50% humidity on a 12h light 12h dark cycle.

### Bacterial stock maintenance

Methods were adapted from (53). *Streptococcus pneumoniae* was grown by shaking in glass vials with 5 mL tryptic soy (TS) broth (Difco TS broth, catalase, streptomycin) at 37°C with 5% CO_2_ overnight. The overgrown culture was diluted in catalase (100 µL) and TS to yield a final volume of 20 mL in a flask and incubated shaking until the OD_600_ ~ 0.4 (about 1 hour). The culture was then diluted again to a final volume of 50 mL, with 150 µL catalase, and incubated until the OD_600_ ~ 0.2 - 0.4 (above 0.5 is no longer in log phase). 5% glycerol was added to the final culture and stored then in 1mL aliquots at -80°C. To use the aliquots, one tube was thawed, spun down at 18,000 g for 5 minutes, the supernatant was removed, and the pellet was resuspended in the desired amount of PBS (50 - 60 µL yields ~ 100,000 CFUs) and serially diluted to yield the appropriate CFU doses. For quantification of CFUs, *S.p.* was plated on TSA agar plates supplemented with 50 mL/L sheep’s blood. *Listeria monocytogenes* (serotype 4b, 19115, (ATCC, VA)) was also grown in batches in brain heart infusion (BHI) medium at 37°C in aerobic condition. Cultures were grown overnight in a flask inoculated with a fresh colony and re-diluted under log phase (below OD_600_ ~ 0.2) and grown up to the desired OD_600_ (~0.4). The entire volume was transferred to a 50mL centrifuge tube for vortexing. Before freezing, a 5% glycerol solution was added to the culture and 1mL aliquots were stored at -80°C. To use the aliquots, one tube was thawed, spun down at 18,000 g for 5 minutes, the supernatant was removed, and the pellet was resuspended in the desired amount of PBS (90 - 100 µL yields ~ 100,000 CFUs) and serially diluted to yield the appropriate CFU doses. For quantification of CFUs, *L.m.* was plated on BHI plates.

### Fly injections, survival and CFUs

Methods were adapted from (53). For injections and immune assays, 5-7-day-old male Oregon R flies were anesthetized with CO_2_ and injected with various CFU doses yielding a total volume of 50 nL precisely using a MINJ-FLY high-speed pneumatic injector (Tritech Research, CA) and individually pulled calibrated glass needles. Flies were injected into the abdomen close to where the thorax meets and slightly ventral from the dorsal-ventral cuticle axis, easily visible below the haltere. Survival studies were carried out for all of the pathogens we tested. After injection of the CFU dose or phosphate buffered saline (PBS) control, flies were placed in vials in groups of 30 with a total of 60 flies per experimental or control group. Flies injected with the human pathogens (*S.p.* and *L.m.)* were kept at 28°C with 50% humidity. The number of dead flies was counted daily, and Kaplan-Meier survival curves were generated with GraphPad Prism software with statistics shown as log-rank analysis (Mantel-Cox). Survival experiments were at least triplicated. CFUs were determined by homogenizing a single infected, or buffer-injected fly in 200 µL of PBS, serially diluted and plated on the appropriate agar plates and incubated overnight. Colonies were counted the next day. At least five flies per condition were homogenized for CFU quantification each time an injection experiment was done to measure time 0 CFUs which are representative of all fly strains. All treatment groups were injected at the same time for each experimental replicate. Using GraphPad Prism software, results are shown as scatter plots with statistical significance analyzed using an unpaired t-test.

### Phenoloxidase activity

Methods adapted from (53). Flies were injected with 10,000 CFUs of *L. monocytogenes* to elicit an immune induced melanization cascade. Phenoloxidase activity was measured as previously described [51,52]. To collect hemolymph, 20-30 flies 6 hours post injection (p.i.) were pricked through the thorax and placed in a pierced 0.5 µL Eppendorf tube and covered with glass beads, then placed inside a 1.5 µL Eppendorf tube containing 30 µL of PBS. Samples were centrifuged at 10,000 g for 20 minutes at 4°C. Using a clear 96-well plate, each well contained 160 µL L-Dopa (3 mg/mL) dissolved in phosphate buffer (37.5% 1 M potassium phosphate, 62.5% 1 M sodium phosphate, pH 6.5), 35 µL of hemolymph sample and 5 µL CaCl_2_ (20 mM). PO activity was measured by kinetic reads at 29°C at 492 nm every minute for 120 min with 5 seconds of shaking between reads. The OD of a blank control was subtracted from all biological values. Experiments were replicated five times with three technical replicates per experiment. Data were plotted as mean+SEM by taking the peak OD value (timepoint ~ 60 min). Statistics shown as an unpaired t-tests done in GraphPad Prism.

### Antimicrobial peptide gene expression - qPCR

Methods adapted from (53). Total RNA was extracted from 15 *S. pneumonia* or *S.p.* plus recombinant protein injected flies 24 hours post-injection using Trizol reagent (Molecular Research Center, Inc; Cincinnati, Ohio) according to the manufacturer instructions. Integrity of RNA was confirmed by observing bands on an agarose gel and concentration was determined by nanodrop. Reverse transcription of RNA was done using ProtoScript II First Strand cDNA synthesis kit (New England BioLabs, NE, E6560L) following the manufacturer protocol, in a MultiGene OptiMax Thermal Cycler (Labnet international, NJ). The qRT-PCR was done with a CFX Connect Bio-Rad system with Perfecta SYBR green supermix (QuantaBio, MA) and gene specific primers for *Defensin, Drosomycin*, *Diptericin, Metchnikowin* and the housekeeping gene *Tubulin* (Integrated DNA Technologies, IA). Cycling conditions for PCR included a denature step at 94 ˚C for 15 seconds, annealing step at 55 ˚C for 30 seconds, and an extension step at 68 ˚C for 1 minute. All steps were conducted for a total of 40 cycles. Fold change was measured according to the ΔΔCT Method. Experiments were carried out with three biological replicates with plots shown as bar graphs with individual points representing each replicate. Statistics shown as One-way ANOVA done in GraphPad Prism.

### Cell culture maintenance

12 ml room temperature of fresh medium [500 mL Schneider’s *Drosophila* medium (Thermo Fisher Scientific, #21720-024-500ML) (Store 4˚C) + 56 mL Fetal bovine serum (FBS; Thermo Fisher Scientific, #10082147, Store at -20˚C or 4˚C) +5.6 mL Penicillin-streptomycin solution (PSS: Thermo Fisher Scientific ^®^, Store at -20˚C or 4˚C)] was added to a new 10 cm plate. A plate of confluent *D. melanogaster* S2 cells were washed by gently adding 5-7mL of room temperature media and gently swirling before aspirating the media. Afterwards another 5-10mL of fresh room temperature media was added and gently pipetted up and down to peel the cells off the bottom of the plate. 3mL of this cell suspension was added to the new plate and gently swirled to help cells attach to the bottom of the plate. The plate was incubated at 25˚C, with humidity of the incubator maintained by autoclaved Milli-Q water. Confluency of 100% is reached within 7 days but repeats of a 1:5 split maintenance is conducted at around ~80% confluency.

### Cell lysis assay

For the cell lysis assay, S2 cells were cultured in a 24-well plate with 0.5 ml medium until cells reached ~75% confluency. After reaching desired confluency, Sc-sPLA_2_ and bee venom sPLA_2_ (from EnzChek™ Phospholipase A2 Assay Kit) were filtered with a 0.45 µm filter before being added to the cell medium. 10 µgs of Sc-sPLA_2_ and 1 µg of bee venom sPLA_2_ were added, and the cell medium was diluted with filtered 20 mM Tris, 300 mM NaCl, and pH 8.0 buffer to a final volume of 0.6 ml (600 µl). Cells were incubated at 25°C for 24 hours. After incubation, the supernatant was aspirated and cells were resuspended with a new volume of 600 µl filtered 20 mM Tris, 300 mM NaCl, and pH 8.0 buffer. 5 µl of cells were then added to 5 µl of trypan blue for a total of 10 µl, and then placed on a dual-chamber slide where percent of live cells were quantified by a Bio-Rad TC20 cell counter. Statistics were shown as one-way ANOVA, with error bars depicting mean with SEM.

### Phrodo phagocytosis

Injections were carried out as previously described for *S. pneumoniae* except with a 4 mg/ml suspension of pHrodo Red *E. coli* BioParticles Conjugate for phagocytosis as a substitute for the bacterial solution. This solution was diluted 1:4 in PBS containing either 5, 10, or 40 ng of Sc-sPLA_2_ immediately prior to injection. A negative control of no protein was injected for analysis along with the 3 different protein doses. 3 flies were injected for each treatment group with a total of 3 biological replicates each. Injected flies were incubated at 28°C with 50% CO_2_ for 1 hour. After incubation, the dorsal side of the abdomen of the flies was imaged with an X-Cite^®^ 120Q fluorescence lamp, and a ZEISS Axiocom 506 Color microscope camera attached to a ZEISS SteREO Discovery V12 microscope at 10x magnification. ImageJ software was used to measure area-normalized corrected total fluorescence of isolated red channels. Statistics were shown as one-way ANOVA, with error bars depicting mean with SEM.

Hemocyte perfusionMethods were adapted from (70). For hemocyte extraction 5–7-day old male Oregon R flies were anesthetized with CO2, washed in 70% ethanol and air dried before cutting the last abdominal segment with a clean scalpel. A fine glass capillary needle was inserted in the anterior part of the thorax and PBS was perfused under air pressure using a MINJ-FLY high-speed pneumatic injector (Tritech Research, CA). Flushed hemocytes were collected onto paraffin film. Flushed hemocytes from 10 flies were pooled together onto paraffin film and taken up with a pipette and placed into a 1.5 mL microcentrifuge tube. Pooled hemocytes were gently mixed by pipetting. 5 µl of hemocytes were added to 5 µl of trypan blue for a total of 10 µl, and then placed on a dual-chamber slide where percent of live cells were quantified by a Bio-Rad TC20 cell counter. Statistics were shown as one-way ANOVA, with error bars depicting mean with SEM.

### *In vitro* metabolomics

The targeted analysis was performed using a QQQ XEVO TQ-XS (Waters Corp., Milford, MA, USA) at the UC Riverside Metabolomics Core. The liquid chromatography-mass spectrometry (LC-MS) autosampler was maintained at 4°C prior to analysis. For the analysis, an injection volume of 2 μL of the extract was used. The separation was performed on the Waters XSelect CSH Phenyl-Hexyl column (3.5 μm, 3.0 × 100 mm (Waters Corp., Milford, MA, USA). The flow rate was maintained at 0.8 mL/min at 30 C. Mobile phase A consisted of ACN/water (95/5, v/v, pH=8.0) containing 25 mM AcNH4 and Mobile Phase B consisted of ACN/water (50/50, v/v, pH=7.5) containing 25 mM AcNH4. The gradient separation method was used as follows: 8 min (0–0.2 min 99% B; 0.2-3.0 min 99% B to 1%B, 3.0-3.8 min 1% B; 3.8-3.9 min 1% B to 99% B; 3.9-8.0 min 99% B. The MS data were acquired in multiple reaction monitoring (MRM) mode. The electrospray ionization was performed in positive ion mode. The source and desolvation temperatures were maintained at 150°C and 600°C, respectively. The desolvation gas was set to 1100 L/h and cone gas to 150 L/hr and the collision gas was set to 0.15 mL/min. All gases used were nitrogen, other than the collision gas which was argon. The capillary voltage was 1.5 kV. The data was normalized for relative abundance against the internal standard (LPC 17:0). Targeted data processing was performed with the open-source Skyline software (69).

### Hemolymph only metabolomics – UCSD

A mix of 26 deuterated internal standards was added to 10uL of hemolymph. Eicosanoids were extracted by solid phase extraction (SPE) using Phenomenex Strata-X polymeric reversed phase columns. Samples were brought to dryness and taken up in buffer A (water/acetonitrile/acetic acid 60/40/0.02, v/v/v). Samples were analyzed using a Waters Acquity UPLC interfaced with an AB Sciex 6500 QTrap instrument. Chromatographic separation was achieved by a step gradient starting with100% buffer A to 100% buffer B (acetonitrile/isopropanol 50/50, v/v) over 5 min. Standard curves were obtained in parallel using identical conditions. Data analysis was performed with Analyst and Mulitquant software packages (71). We monitored 159 MRMs.

### Statistics

All statistics were done with GraphPad Prism 9.1.0 for Mac. Statistical significance indicated with asterisks indicating the following p-value cut offs: 0.05-0.033*, 0.033-0.002**, 0.002-0.0001*** and <0.0001****. Data with an n-value more than 10 was checked for normality and lognormality and the appropriate tests were performed based on these results. For data with and n-value less than 10, normal Gaussian distribution was assumed. Survival curves were analyzed by plotting one treatment group and its control to measure the curve comparison.

## Data availability statement

The raw data supporting the conclusions of this article will be made available by the authors, without undue reservation.

## Author contributions

SCP carried out toxicity and immunomodulatory studies, conceptualized, planned, and carried out lipid metabolite studies, and wrote and designed the manuscript. OKO designed plasmid construction, produced and purified recombinant protein for the study, performed enzymatic and lipidomic protein activity assays, assisted in the phagocytosis experiments, and wrote and designed the manuscript. PA performed fly injection experiments for hemocyte counts and immunomodulatory studies, and contributed to the writing of the manuscript. SN, SM-B, and IC assisted in toxicity, immunomodulatory, and mass spectrometry assays, and in maintaining and scoring flies. KA assisted in lipid metabolite studies. AB helped design, optimize, and carry out the mass spectrometry activity assays. HD assisted in recombinant protein production and optimization of protein purification. AD secured funding, managed the project, and assisted in the development of the manuscript. All authors contributed to the article and approved the submitted version.
