# Supplementary material for: Parasitic nematode secreted phospholipase A2 suppresses cellular and humoral immunity by targeting hemocytes in Drosophila melanogaster
Source: Front Immunol. 2023 Mar 15;14:1122451. doi: 10.3389/fimmu.2023.1122451 (PMC10050561; doi:10.3389/fimmu.2023.1122451)
Supplement: Supplementary file 1 [file DataSheet_1.pdf]

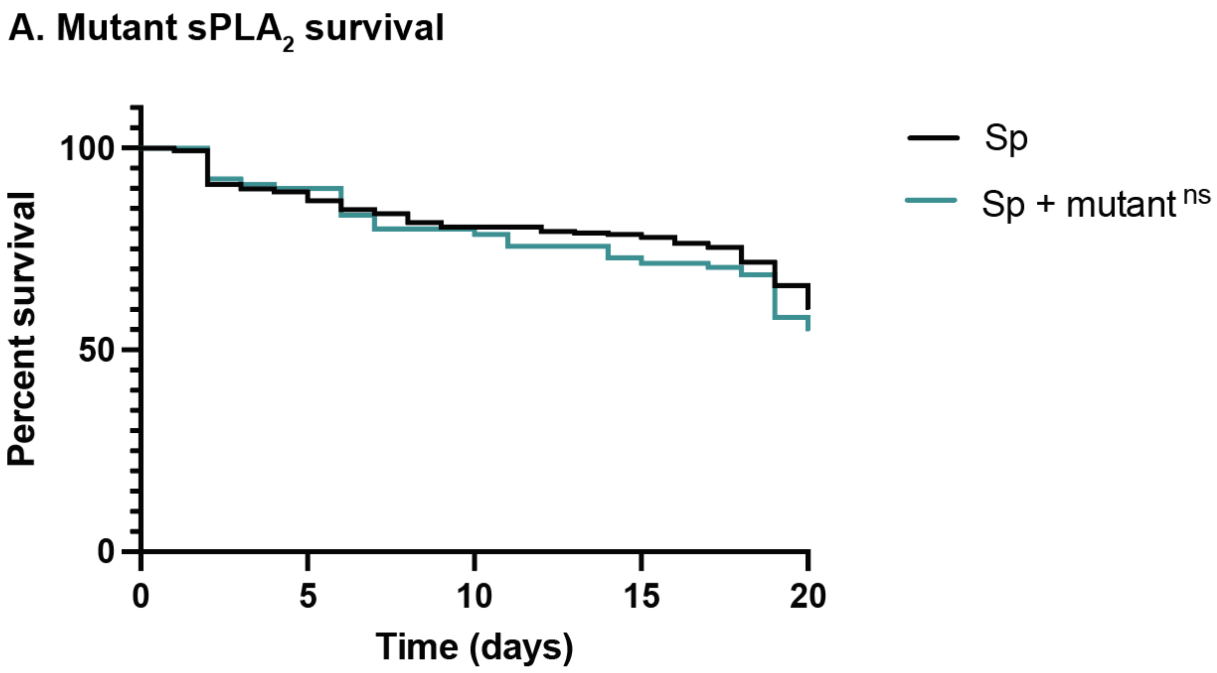

Supplemental Figure 1: Inactive mutant $sPLA_2$ does not show an immunomodulatory effect. Flies were injected with 2,000 cells S.p. or 2,000 cells S.p. plus mutant $sPLA_2$ and survival was monitored for 20 days. There is no significant difference between the two treatment groups. Each treatment group represents at least 180 flies on the Kaplan Meier graph with significance shown as log-rank Mantel-Cox) test.

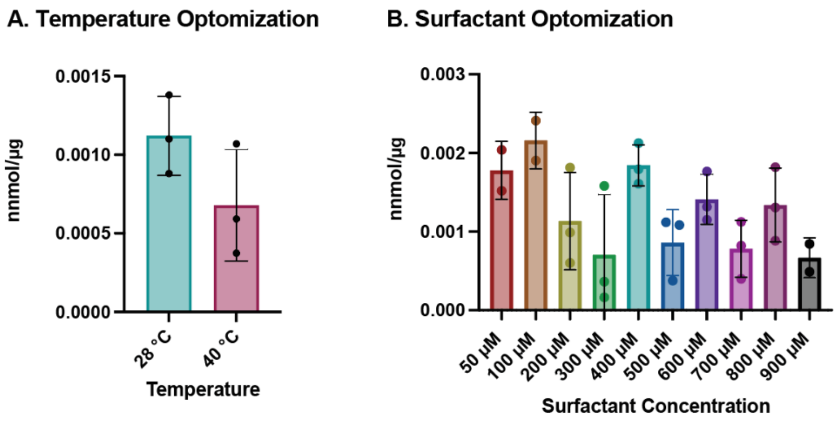

Supplemental Figure 2. A) Enzyme activity of Sc-$sPLA_2$ towards 100 µM of PLPC at two different temperatures (28°C and 40°C). B) Enzyme activity of Sc-$sPLA_2$ towards 100 µM of PLPC with varying concentrations of surfactant. Negative control values were subtracted as background from each reaction (A and B). Experiments were done in either duplicate or triplicate. All statistics shown as unpaired t-test, error bars depict mean with SEM.

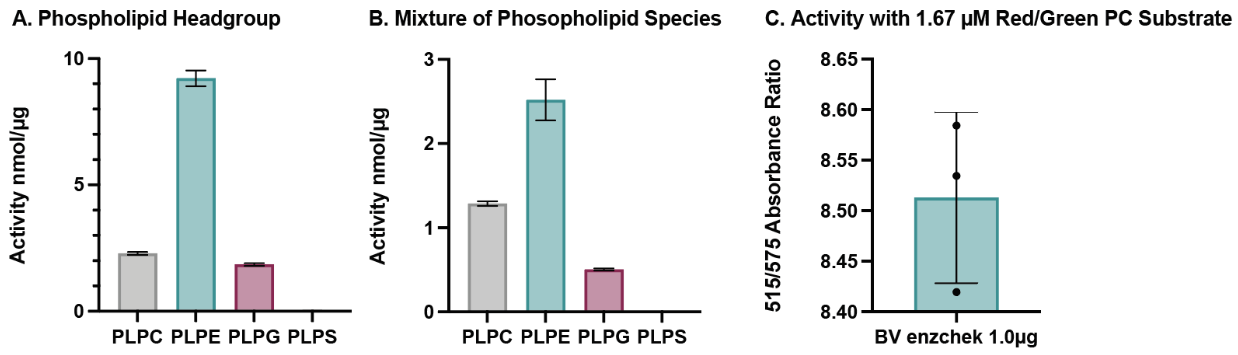

Supplemental Figure 3. A) Enzymatic activity of Bee venom $sPLA_2$ towards 100 µM of PLPC, PLPE, PLPG and PLPS. B) Enzymatic activity of Bee venom $sPLA_2$ towards 100 µM mixture (20 µM each) of PLPC, PLPE, PLPG and PLPS. Negative control values were subtracted as background from each reaction (A and B). C) *In vitro* activity data of Bee venom $sPLA_2$ at 1.0 µg. Fluorescent emission intensity was measured at 515 and 575 nm and recorded as a ratiometric value. Negative control was subtracted as background from both absorbance values before calculating the ratio. Substrate used was a Red/Green Bodipy labeled PC. Experiments were done in triplicate. All statistics shown as unpaired t-test, error bars depict mean with SEM.

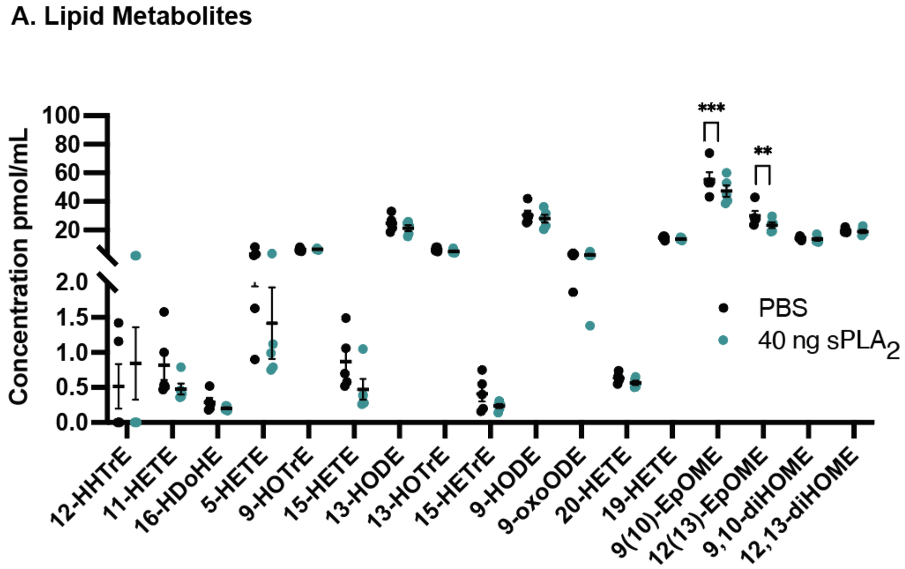

Supplemental Figure 4. Injection of recombinant Sc-$sPLA_2$ reduces 9,(10)-EpOME and 12,(13)-EpOME in fly hemolymph 12 hours post injection. Flies were injected with PBS or 40 ng $sPLA_2$ and pooled hemolymph was analyzed for downstream lipid metabolites 12 hours post injection. 9(10)-EpOME and 12,(13)-EpOME showed a significant reduction in the 40 ng protein group. Experiments were repeated 5 times with 200 flies per treatment group. Out of 131 metabolites, 17 were detected in fly hemolymph samples. Error bars show mean + SEM with statistics shown as unpaired t-test.
